# Paul Farmer: A Pioneer of Global Health, Health Equity, and Health Advocacy

**DOI:** 10.7759/cureus.70724

**Published:** 2024-10-02

**Authors:** Hanna Schindler, Michelle Wallen

**Affiliations:** 1 Research, Orlando College of Osteopathic Medicine, Winter Garden, USA; 2 Emergency Medicine, UCF Lake Nona Hospital, Orlando, USA

**Keywords:** global health care, global public health, health care disparity, health-care equity, health-care policy, public health, public health aspects and anthropology, public health care

## Abstract

Paul Farmer, a distinguished physician and anthropologist known by many as a pioneer in global health endeavors, made a profound impact on the landscape of healthcare. Through his innovative approaches to healthcare delivery and his unwavering commitment to social justice and health equity, he changed the landscape of healthcare on a global scale. He was a Kolokotrones University Professor, Chair of the Department of Global Health and Social Medicine at Harvard Medical School, Chief of the Division of Global Health Equity at Brigham and Women's Hospital in Boston, Chancellor of the University of Global Health Equity in Rwanda, and Chief Strategist and Co-founder of Partners In Health (PIH). PIH is an international non-profit organization that embarked on research and healthcare advocacy initiatives, providing direct healthcare services for those in resource-limited communities worldwide. Farmer, through his development of innovative and heartfelt approaches to healthcare delivery and social justice medicine, exemplified how integrating community engagement and social determinants of health can lead to transformative healthcare solutions and ultimately improve patient experiences. This vignette explores Farmer’s early life, educational journey, and major contributions to medicine and social justice in healthcare, emphasizing the enduring impact of his work on public health and future generations of medical professionals.

## Introduction and background

In a world where healthcare access is often determined by socioeconomic status, Paul Farmer was dedicated to dismantling barriers to healthcare and advocating for populations who face structural violence on a global scale [1]. Born on October 29, 1959, in North Adams, Massachusetts, Farmer was raised for a short time in Alabama and spent his later childhood years in Florida. He had a somewhat unconventional childhood and moved frequently with his family due to his father’s job as a commercial fisherman. The family undertook homesteading on a houseboat and later in a converted bus without running water, where Farmer spent many years as a child. Although alternative, his upbringing and family dynamic greatly emphasized education and community service. Thus, Farmer had early experiences with volunteering that surely shaped his passion for social justice. Early experiences working closely with migrant workers would foreshadow the work he would undertake in Haiti later in his career. He excelled in grade school and went on to attend Duke University, where he graduated summa cum laude with a Bachelor of Arts in Medical Anthropology in 1982. During his time there, he volunteered in Haitian migrant worker camps, where he developed deep relationships with many Haitian farm workers. Through hearing about their experiences, he decided to embark on work in Haiti's Central Plateau, focusing on helping incorporate modern healthcare practices in their communities. Once returning from Haiti, he completed a post-graduate fellowship at the University of Pittsburgh and soon applied to Harvard Medical School, one of two institutions in the United States to offer a joint-degree program in Medicine and Medical Anthropology at the time. While in the application cycle for medical school, he traveled to Haiti, where he worked in public health clinics, mastered the Creole language, and learned more about one of the most resource-strained countries in the world. His belief that "some lives matter less is the root of all that is wrong with the world" has guided his work throughout his career [2]. He went on to attend Harvard Medical School, earning an MD and a PhD in Medical Anthropology in 1990, and thereafter created a lifetime of work that greatly contributed to global health equity and patient advocacy.

## Review

Birth of Partners In Health (PIH)

While attending Harvard University for medical school, Farmer returned to Haiti frequently to continue his work in medical outreach and support clinics there [2]. He wanted to solidify his efforts through the creation of an organization, which ultimately led to his co-founding of PIH in 1987 with his colleagues from Harvard: Jim Yong Kim, Ophelia Dahl, Thomas J. White, and Todd McCormack. This organization was developed based on the belief that healthcare is a right, not a privilege. Farmer and PIH implemented innovative models of healthcare that integrated social support with medical treatment. At the time, this was not commonplace practice.

Farmer’s work with PIH grew to extend beyond Haiti, and he made significant contributions in Rwanda in the wake of the Rwandan genocide and thereafter. During the genocide, PIH helped establish a comprehensive treatment program that trained local healthcare workers to deliver care and treatment for diseases like HIV, AIDS, and tuberculosis. They provided free access to medications and established programs for a directly observed therapy (DOT) care model, which ensures adherence to treatment methods [3].

Farmer’s work in Haiti and Rwanda exemplifies the importance of community engagement in public health initiatives. By stating, “Health care is a right, and it cannot be guaranteed without the active participation of the communities it is meant to serve,” he underscores the necessity of incorporating local knowledge and resources into healthcare delivery [4]. This participatory approach has inspired many healthcare education systems and healthcare providers to adopt similar models in their teachings and practices.

In addition to HIV and AIDS treatment model development and social medicine implementation, PIH has advocated for addressing non-communicable diseases in low-income countries. Farmer recognized that effective healthcare must encompass a wide range of health issues, especially in settings where patients often face multiple health challenges simultaneously. His holistic approach to healthcare has encouraged policymakers to rethink their strategies for managing health in resource-limited environments [5].

Major contributions in healthcare

One of Farmer’s first major anthropological connections to healthcare resided in the “Accompaniment Model,” which was a groundbreaking approach that paired patients with community health workers. This model recognizes that socioeconomic factors, such as poverty and lack of education, often prevent individuals from accessing the care they need. Farmer initially borrowed the idea from liberation theology and applied it to medicine and medical care [6]. He turned it into the guiding practice for clinical work among poor HIV and AIDS patients in Haiti, those suffering from multidrug-resistant TB in Peru, and Ebola patients in West Africa. By providing support and education, trained community health workers empower patients to navigate healthcare systems, adhere to treatment, and improve their overall health outcomes [7]. This model is truly the birth of patient advocacy and informed patient care models that are used in many healthcare systems today. By actively engaging patients and addressing their unique challenges, Farmer's implementation of the Accompaniment Model in healthcare fostered a sense of agency and partnership, encouraging patients to take ownership of their health and advocate for increased quality of care through this partnership. Moreover, it highlights the importance of integrating social justice into healthcare practices, ensuring that marginalized populations receive not only medical attention but also the social support necessary to thrive. As such, the Accompaniment Model exemplifies the critical intersection of health advocacy and community empowerment, reshaping how healthcare providers approach patient care in resource-limited settings and beyond through systemic change.

Broader implications for global health

Farmer's contributions have fundamentally transformed the landscape of global health, demonstrating that high-quality care can be delivered in resource-limited settings. His work has influenced healthcare delivery models worldwide, emphasizing the importance of addressing social determinants of health and fostering community involvement. By advocating for the rights of marginalized populations, he has inspired a movement toward more compassionate and holistic healthcare practices. Many of Farmer’s books, including but not limited to *Pathologies of Power: Health, Human Rights, The New War on the Poor, To Repair the World: Paul Farmer Speaks to the Next Generation, Global Health in Times of Violence,* and *Reimagining Global Health*, articulate his vision for a broader global health standard, linking health disparities to broader social injustices. He asserts that health cannot be separated from human rights, emphasizing that effective public health efforts must address the root causes of inequality [7]. Many of the insights explored in his books and the strategies implemented in his healthcare efforts have prompted a reevaluation of public health strategies, advocating for comprehensive approaches that prioritize equity and accessibility on a global scale. In addition to his writings, Farmer has been involved in numerous public health initiatives and advisory boards, aiding in shaping policy discussions on global health. His work and expertise with organizations like the World Health Organization and the United Nations have highlighted the need for collaborative approaches to address global health challenges, further solidifying his influence on public health policy [6].

Research contributions

As PIH continued to grow, they conducted a variety of research studies focused on improving healthcare delivery and addressing the social determinants of health in the communities they serve. These studies not only contribute to the scientific understanding of health interventions in low-resource settings but they have also been applied to other healthcare settings to ensure top-quality care. The findings further contribute to the broader field of global health (Table 1) [8].

**Table 1:** Summary of research topic areas of Partners In Health (PIH). Table credit: [8]

Topic area	Research
HIV/AIDS treatment and care	Research on the effectiveness of antiretroviral therapy (ART) in resource-limited settings, examining factors that influence patient retention and adherence to treatment.
	Studies evaluating the impact of community-based approaches to HIV prevention, including the role of peer educators and community health workers.
Tuberculosis	Multidrug-resistant tuberculosis (MDR-TB): Research on the treatment outcomes of MDR-TB patients, assessing the efficacy of directly observed therapy (DOT) and community-based care models.
	Integration of TB and HIV services: Studies exploring the benefits of integrated care for patients co-infected with TB and HIV, leading to improved health outcomes.
Maternal and child health	Research on the effectiveness of community health worker programs in improving maternal health outcomes, such as prenatal care access and safe delivery practices.
	Evaluations of childhood vaccination programs and the impact of community health initiatives on reducing child mortality rates.
Social determinants of health	Studies investigating how socioeconomic factors, such as poverty, education, and housing, impact health outcomes in the populations served by Partners In Health (PIH). This research supports advocacy for policy changes to address health disparities.
Mental health integration	Research examining the effectiveness of integrating mental health services into primary care settings, particularly in post-conflict regions and low-resource areas.
Ebola response	Evaluation of the effectiveness of treatment protocols and community engagement strategies during the Ebola outbreak in West Africa, providing insights into best practices for future outbreaks.
Health system strengthening	Studies on the impact of training local healthcare workers and building healthcare infrastructure, focusing on sustainable models of care that can be maintained long-term.
Global health policy	Research that informs global health policies, advocating for increased funding and resources for health systems in low-income countries, and addressing barriers to care.

Mentorship and influence

Farmer’s impact extends beyond healthcare delivery, as he has made significant contributions to education and mentorship, which more widely impact healthcare advocacy as a model. This allows his students and partners to better understand and develop a structured approach to empower patients and communities in navigating the healthcare system. As a professor at Harvard Medical School and the founding chair of the Department of Global Health and Social Medicine in 2010, Farmer has trained and inspired countless students and healthcare professionals. His teachings emphasize the importance of understanding the social context of health and the need for community involvement in healthcare delivery, establishing and contributing to the healthcare advocacy model that continues to be a progressive approach in healthcare. Many of Farmer's mentees have adopted his models of care, carrying forward his legacy of compassion and social responsibility in healthcare delivery through patient advocacy. He encouraged his students to view their work as part of a larger movement for social change, focusing on the duty to address the social factors that shape health outcomes and the willingness to challenge the status quo [6]. His commitment to mentorship has fostered a new generation of healthcare leaders dedicated to social justice and health equity [9].

## Conclusions

Paul Farmer’s legacy as a pioneer of global health and a champion for health equity is undeniable. His innovative approaches to healthcare delivery, unwavering advocacy for marginalized populations, and dedication to mentorship have left an indelible mark on the field of medicine and global health through patient advocacy. As future healthcare professionals strive to uphold Farmer's principles, they are reminded of the profound impact that dedication, compassion, and social justice can have on human health. His passion inspires current and future healthcare professionals to continue the fight for a more just healthcare system, recognizing that health is a fundamental right for all. Paul Farmer passed away on February 21, 2022, due to a sudden cardiac event while sleeping in his apartment at a university he helped establish in Rwanda.
